# The Contribution of Plasma Urea to Total Osmolality During Iatrogenic Fluid Reduction in Critically Ill Patients

**DOI:** 10.1093/function/zqab055

**Published:** 2021-10-29

**Authors:** Sandra Nihlén, Robert Frithiof, Jens Titze, Rafael Kawati, Johan Rasmusson, Christian Rylander, Andreas Pikwer, Markus Castegren, Anton Belin, Michael Hultström, Miklos Lipcsey

**Affiliations:** Department of Surgical Sciences, Anesthesiology and Intensive Care, Uppsala University, SE-751 05 Uppsala, Sweden; Department of Surgical Sciences, Anesthesiology and Intensive Care, Uppsala University, SE-751 05 Uppsala, Sweden; Programme in Cardiovascular and Metabolic Disorders, Duke-NUS Medical School, Singapore 169856, Singapore; Division of Nephrology and Hypertension, Friedrich-Alexander University Erlangen-Nuremberg, 91012 Erlangen, Germany; Division of Nephrology, Duke University Medical Center, Durham, NC 27710, USA; Department of Surgical Sciences, Anesthesiology and Intensive Care, Uppsala University, SE-751 05 Uppsala, Sweden; Department of Anesthesiology and Intensive Care, Gävle County Hospital, SE-801 87 Gävle, Sweden; Department of Anaesthesiology and Intensive Care Medicine, Institute of Clinical Sciences, Sahlgrenska Academy, University of Gothenburg, SE-413 45 Göteborg, Sweden; Centre for Clinical Research Sörmland, Uppsala University, SE-631 88 Eskilstuna, Sweden; Centre for Clinical Research Sörmland, Uppsala University, SE-631 88 Eskilstuna, Sweden; Perioperative Medicine and Intensive Care, Karolinska University Hospital, and FyFa, Karolinska Institutet, SE-171 77 Stockholm, Sweden; Department of Surgical Sciences, Anesthesiology and Intensive Care, Uppsala University, SE-751 05 Uppsala, Sweden; Department of Surgical Sciences, Anesthesiology and Intensive Care, Uppsala University, SE-751 05 Uppsala, Sweden; Integrative Physiology, Department of Medical Cell Biology, Uppsala University, SE-751 23 Uppsala, Sweden; Department of Surgical Sciences, Anesthesiology and Intensive Care, Uppsala University, SE-751 05 Uppsala, Sweden; Hedenstierna Laboratory, CIRRUS, Department of Surgical Sciences, Anesthesiology and Intensive Care, Uppsala University, SE-751 05 Uppsala, Sweden, and Akademiska sjukhuset, SE-751 85 Uppsala, Sweden

**Keywords:** critical care, fluid therapy, water–electrolyte balance, osmolar concentration, urea

## Abstract

Hyperosmolality is common in critically ill patients during body fluid volume reduction. It is unknown whether this is only a result of decreased total body water or an active osmole-producing mechanism similar to that found in aestivating animals, where muscle degradation increases urea levels to preserve water. We hypothesized that fluid volume reduction in critically ill patients contributes to a shift from ionic to organic osmolytes similar to mechanisms of aestivation. We performed a post-hoc analysis on data from a multicenter observational study in adult intensive care unit (ICU) patients in the postresuscitative phase. Fluid, electrolyte, energy and nitrogen intake, fluid loss, estimated glomerular filtration rate (eGFR), and estimated plasma osmolality (eOSM) were registered. Contributions of osmolytes Na^+^, K^+^, urea, and glucose to eOSM expressed as proportions of eOSM were calculated. A total of 241 patients were included. eOSM increased (median change 7.4 mOsm/kg [IQR−1.9–18]) during the study. Sodium's and potassium's proportions of eOSM decreased (*P* < .05 and *P* < .01, respectively), whereas urea's proportion increased (*P* < .001). The urea’s proportion of eOSM was higher in patients with negative vs. positive fluid balance. Urea's proportion of eOSM increased with eOSM (*r* = 0.63; adjusted for eGFR *r* = 0.80), but not nitrogen intake. In patients without furosemide and/or renal replacement therapy (*n* = 17), urea’s proportion of eOSM and eOSM correlated strongly (*r* = 0.92). Urea’s proportion of eOSM was higher in patients not surviving up to 90 d. In stabilized ICU patients, the contribution of urea to plasma osmolality increased during body water volume reduction, statistically independently of nitrogen administration and eGFR. The shift from ionic osmolytes to urea during body fluid volume reduction is similar to that seen in aestivating animals.

ClinicalTrials.org Identifier: NCT03972475.

## Graphical Abstract

**Graphical Abstract:**
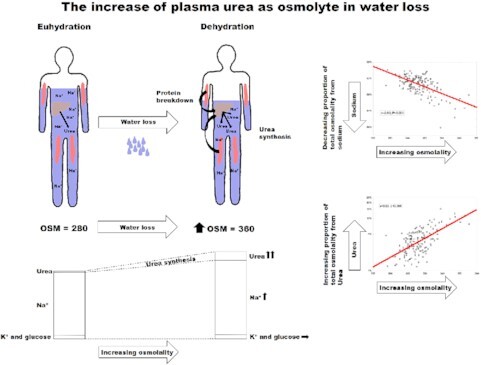

## Introduction

The goal of fluid administration in the intensive care unit (ICU) is to sustain homeostasis and to prevent and treat shock and multiple organ dysfunction. At the same time, iatrogenic fluid overload is linked to complications.^1,2^ For many years, focus has been on the administration of excessive amounts of resuscitative fluids and its negative effect on patient outcome.^3,4^ Fluid volume reduction is commenced in ICU patients after the initial resuscitation phase.^5^ We have shown that almost all stabilized patients were treated with fluid volume reduction at some point during their ICU stay ^6^ and estimated plasma osmolality (eOSM) increased during the fluid volume reduction phase.^6^

Osmolality plays an important role in extracellular and intracellular water distribution and mainly depends on the serum concentrations of sodium, potassium, anions, glucose, and urea.^7^ Dehydration is intra and extracellular net loss of water^8^ that is manifested in ICU patients with negative fluid balance and increased osmolality. Fluid volume reduction, especially with diuretics, increases free water losses,^9^ thus causes iatrogenic dehydration. There is a consistent association between hyperosmolality and mortality in some populations.^10^ In critically ill patients with serum osmolarity over 300 mmol/L, increased mortality has been reported in patients with cardiac, cerebral, vascular, and gastrointestinal admission diagnoses, but not in those with respiratory disease.^11,12^

Traditionally, increased osmolality induced by fluid volume reduction is considered to be a result of increased water loss alone. Yet, recent reports suggest that there is a coordinated response to dehydration in rodents and humans that include a gradual switch from ionic osmolytes in form of sodium, to organic osmolytes in form of glucose and urea.^13,14^ The postresuscitative phase in critically ill patients, where water loss and nutrition below metabolic needs are common,^6^ has previously been compared to torpor,^15^ a condition related to aestivation. Moreover, physiological adaptation to body water loss is characterized by catabolic exploitation of muscle energy and nitrogen for body water conservation. Recent evidence suggests that this adaptive water conservation response pattern, which has been termed *aestivation* in zoology, may not be restricted to amphibians and fish, but also occurs in mammals.^16^ Experimental studies indicate that chronic body water loss triggers a hepato–renal gluconeogenic/ureagenic response for organic solute production to stabilize the intra and extracellular volume.^13,17,18^ An increase in plasma urea solute concentration to maintain serum osmolality is a simple clinical marker of this systemic aestivation-like systemic adaptive water conservation response.

We hypothesized that water conservation metabolism exists in ICU patients during the post resuscitative fluid volume reduction phase, ie that dehydration could lead to a shift from ionic osmolytes to nonionic organic osmolytes. Such a water conservation mechanism could bear similarities to that seen in aestivating animals. We conducted a posthoc study on data from a multicenter study on ICU patients in the post resuscitative phase to test this hypothesis.^6^

Our primary outcome was to assess whether plasma urea's proportion of estimated plasma osmolality (Prop_Urea/eOSM_) is associated with estimated plasma osmolality (eOSM). Our secondary outcome was to study the predictors of Prop_Urea/eOSM_ as well as the relationship between Prop_Urea/eOSM_ and 90-day mortality.

## Materials and Methods

### Ethics

The retrospective observational study was approved by the Regional Research Ethics Committee of Uppsala, Sweden (Dnr 2018/170), conducted in accordance with the Helsinki declaration and its subsequent revisions and registered in ClinicalTrials.org (Identifier: NCT03972475). Reporting follows the STROBE Statement.

### Design

In this posthoc analysis on data from our previous retrospective observational study^6^ on stable ICU patients in the post resuscitative phase, we included adult patients >18 yr of age who spent at least 7 d in a participating ICU during 2018. Patient inclusion was consecutive at all participating ICUs and terminated when reaching our preset goal number of patients. A total of six mixed surgical and medical ICUs in Sweden participated in the study: Four university hospital ICUs (Karolinska University Hospital Solna and Huddinge sites, Uppsala University Hospital, Sahlgrenska University Hospital) and two regional hospital ICUs (Gävle Hospital and Mälarsjukhuset, Eskilstuna).

### Data

Demographic data such as age, sex, diagnosis, and Simplified Acute Physiology Score 3 (SAPS 3)^19,20^ were collected. Data were collected on fluid input and output during ICU days 3–7. The time interval specially chosen not to include the first 48 h when patients more often are hemodynamically unstable and are often treated with resuscitation fluids.

Data were also registered on weight and fluid balance and laboratory tests such as plasma sodium, plasma potassium, plasma urea, Cystatin C estimated glomerular filtration rate (eGFR), and 90-day mortality. We defined eGFR as normal above 60 mL/min and decreased if 59 mL/min or lower.^21^ The amount and type of fluids given to patients and all body fluid losses due to urinary output, renal replacement therapy (RRT), gastrointestinal losses, drainage losses, and calculated perspiration were registered. Based on the type and amount of enteral and parenteral fluids, we calculated daily administration of energy (kCal) and nitrogen for each patient. Insensible losses were estimated to 10 mL/kg/d for all patients as this is the arbitrary formula used in some of the ICUs in our study. The amount of furosemide, the most commonly used diuretic used in Swedish ICUs, was also registered. Electrolytes and glucose were measured on blood gas analyzers in the units, whereas urea was measured in each hospital's central laboratory. Serum osmolality was estimated using the formula: eOSM = 2Na^+^+2K^+^+urea + glucose (mOSM/kg).^7^ We calculated how much the osmolites Na^+^, K^+^, urea, and glucose contributes to the estimated total serum osmolality expressed as proportion of the estimated total plasma osmolality for these (Prop_Na+/eOSM_, Prop_K+/eOSM_, Prop_Urea/eOSM_, and Prop_Glucose/eOSM_, respectively). Data were registered in the OpenClinica database (version: 3.11, Waltham, MA).

### Statistics

The sample size was calculated for the original study.^6^ The proportion of missing data for key variables was less than 10%, but for the composite variables Prop_Na+/eOSM_, Prop_K+/eOSM_, Prop_Urea/eOSM_, and Prop_Glucose/eOSM_ missing data was 28%. Given the explorative nature of the study and that data was missing at random, calculations were based on the original data set. However, data for these variables were also imputed and a sensitivity analysis was performed with imputed data for the main outcome.

Data were assessed for normality and data with log-normal distribution were log-transformed. Continuous data is presented as mean ± SD or median (IQR) unless stated otherwise. Frequencies are presented as absolute numbers of total participants (% of study population). Correlations were assessed with parametric univariate models or multivariate models adjusting for eGFR (as surrogate for urea clearance) or furosemide dose. Residuals were assessed for normality. Correlations with correlation coefficients *r* > 0.5 were considered as strong associations. We used *T*-test for comparisons of quantitative continuous variables between two groups. To assess differences for repeated measurements between groups and over time we used a general linear model (ANOVA III). Logistic regression with was used to calculate odds ratios. Statistica (Version 13.5, Tibco Software Inc., Palo Alto, CA) was used for the calculations. *P* < .05 was considered significant.

## Results

We included 241 patients in the study, 62% of them were men. Median age was 63 (51–73) yr and median SAPS3 score at admission was 69 (59–82). On ICU day 3, 83% received mechanical ventilation and 77% were treated with vasopressor therapy on the same day, while 34% of the patients were given CRRT at some time during the study period. Mortality at 90 d post ICU admission was 30% in the cohort. The most common diagnoses were septic shock (11.3%), cardiac arrest (6.6%), unspecified bacterial pneumonia (5.9%), unspecified respiratory insufficiency (5.9%), severe acute respiratory distress syndrome (5.9%), and multiple traumatic injuries (5.9%). Further demographics have been presented previously.^6^

Median plasma sodium (137 (134–140) to 141 (137–144) mmol/L), plasma urea (10.1 (5.7–17) to 11.1 (7.6–16) mmol/L, and eGFR (49 (27–70) to 63 (38–88) mL/min) increased from ICU days 3–7, while potassium (4.1 (3.9–4.2) to 4.2 (3.9–4.4)) and glucose (7.8 (6.9–9.6) to 7.8 (7.0–9.3) mmol/L) remained unchanged. Median plasma creatinine decreased from 111 (72–188) to 91 (62–145) µmol/L (Table S1). The total median fluid administration ICU days 3–7 was 15 529 (13 163–18 111) mL, while the total median fluid loss was 18 712 (15 781–22 398) mL.^6^ eOSM increased from 303 (297–310) on ICU day 3 to 311 (303–319) mOSM/kg on ICU day 7 as previously reported, with a median change in eOSM of 7.4 (IQR −1.9–18) ICU days 3–7.^6^

There was a strong negative correlation between plasma urea and eGFR (Table 1). Plasma urea increased with energy intake and negative fluid balance but these associations were not strong. Nitrogen intake and fluid administration did not correlate with plasma urea. Correlations were similar for Prop_Urea/eOSM_ (Table 2).

**Table 1.** Correlation coefficients from univariate linear regression analysis of the plasma urea and predictors. *P < .05, **P < .01, and ***P < .001.

	ICU day 3	ICU day 4	ICU day 5	ICU day 6	ICU day 7
Urea vs. nitrogen administration	−0.03	−0.03	−0.06	0.02	0.07
Urea vs. fat administration	0.13	0.22**	0.06	0.23**	0.19*
Urea vs. energy administration	0.06	0.21**	−0.10	0.22**	0.19*
Urea vs. eGFR	−0.63***	−0.61***	−0.59***	−0.60***	−0.62***
Urea vs. fluid balance	−0.02	−0.13	−0.21*	−0.10	0.09
Urea vs. fluid administration	−0.04	−0.08	−0.06	−0.04	0.06

**Table 2.** Correlation coefficients from univariate linear regression analysis of the proportion of urea of estimated serum osmolality (Prop_Urea/eOSM_) and predictors. *P < .05, **P < .01, and ***P < .001.

	ICU day 3	ICU day 4	ICU day 5	ICU day 6	ICU day 7
Prop_Urea/eOSM_ vs. nitrogen administration	−0.09	−0.05	−0.06	0.02	0.11
Prop_Urea/eOSM_ vs. fat administration	0.15	0.21**	0.05	0.19*	0.19*
Prop_Urea/eOSM_ vs. energy administration	0.06	0.20**	0.08	0.19*	0.19*
Prop_Urea/eOSM_ vs. eGFR	−0.64***	−0.62***	−0.60***	−0.60***	−0.63***
Prop_Urea/eOSM_ vs. fluid balance	−0.05	−0.13	−0.19*	−0.09	0.07
Prop_Urea/eOSM_ vs. fluid administration	−0.08	−0.06	−0.05	−0.02	0.04

**Figure 1.**
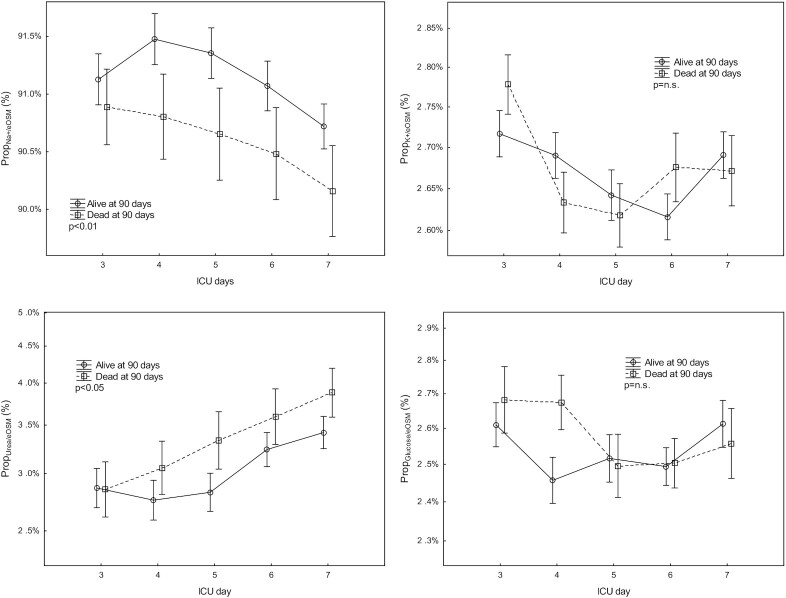
The evolution of the proportions (Prop) of plasma sodium, potassium, urea, and glucose of total estimated serum osmolality (eOsm) vs. eOsm on days 3 and 7 of intensive care (ICU) in survivors and nonsurvivors at 90 d. Mean ± SEM.

Prop_Na+/eOSM_ and Prop_K+/eOSM_ decreased (*P* < .05 and *P* < .01 respectively), while Prop_Urea/eOSM_ increased (*P* < .001) from ICU days 3–7 (Figure 1). Prop_glucose/eOSM_ was unchanged during the same period.

**Figure 2.**
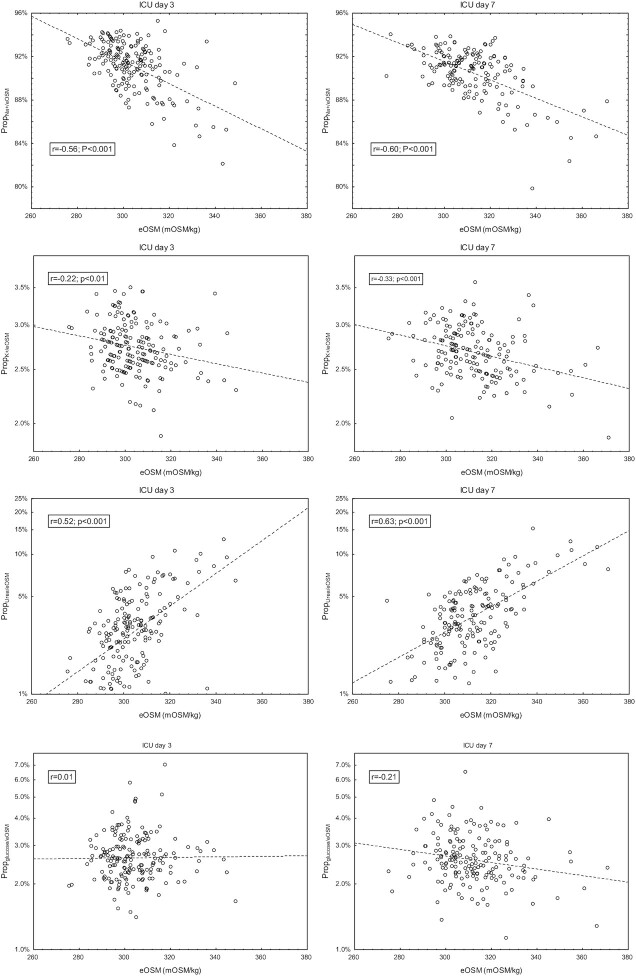
Correlation between the proportions (Prop) of plasma sodium, potassium, urea and glucose of total estimated serum osmolality (eOsm) vs. eOSM on days 3 and 7 of intensive care (ICU).

Prop_Na+/eOSM_ and Prop_Urea+/eOSM_ had the highest negative respectively positive correlation coefficients to eOSM (Figure 2), while Prop_K+/eOSM_ had a weak negative correlation coefficients to eOSM on ICU days 3 and 7. Prop_Glucose/eOSM_ did not correlate to eOSM. In the patients with eOSM below 10th percentile the correlation between eOSM and Prop_Urea+/eOSM_ ICU day 7 was *r* = 0.62.

**Figure 3.**
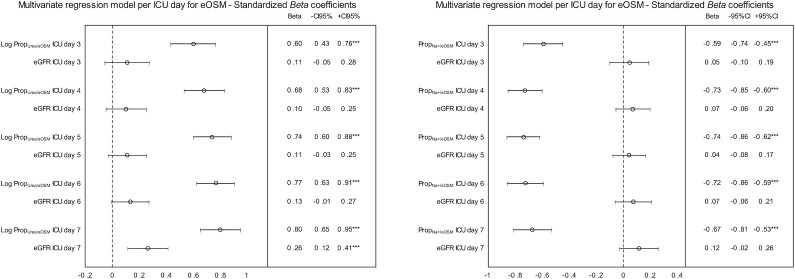
The evolution of standardized *beta* regression coefficients with 95% confidence intervals for estimated serum osmolality (eOsm) vs. proportions of urea of total estimated serum osmolality (Prop_Urea/eOSM_) and estimated glomerular filtration rate (eGFR) as well as for eOSM vs. proportions of sodium of total estimated serum osmolality (Prop_Na+/eOSM_)and eGFR from ICU days 3–7. eOSM was the dependent variable and Prop_Urea/eOSM_ and eGFR as well as Prop_Na+/eOSM_ and eGFR were predictors in multivariate linear regressions model for each ICU day.

When patients were grouped according to normal (*n* = 122) or decreased eGFR (*n* = 101), correlation coefficients for eOSM vs. Prop_Urea+/eOSM_ were *r* = 0.61 and *r* = 0.72 respectively on ICU day 7. In a multivariate regression model for Prop_Na+/eOSM_ and Prop_Urea+/eOSM_ were associated with eOSM while eGFR was not (Figure 3). Total urine output ICU days 3–7 did not correlate to Prop_Urea+/eOSM_ in patients not receiving CRRT (*r* = 0.10, *P* = .29).

**Figure 4.**
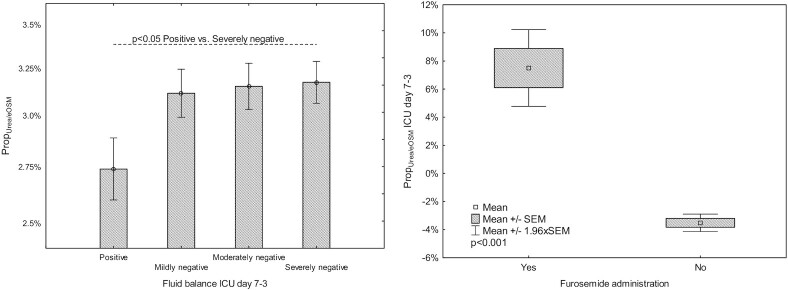
(A) Proportions of urea of total estimated serum osmolality (Prop_Urea/eOSM_) in patient groups according to fluid balance ICU days 7–3. Fluid balance was grouped into positive and tertiles of negative balance. Mean ± SEM. (B) Prop_Urea/eOSM_ in patients with and without furosemide treatment.

Total fluid balance on ICU days 7–3 was grouped into positive and tertiles of negative balance termed mild, moderate, and severe negative fluid balance with <0,  <−3153, and <−5467 mL as limits. Patients with positive fluid balance ICU days 7–3 had lower Prop_Urea+/eOSM_ during this period than those with severe dehydration (Figure 4A, *P* < .05).

During ICU days 3–7, 177 patients (73%) were treated with furosemide on one or more occasions. We assessed the effect of the cumulative furosemide dose ICU days 3–7, and there was a strong correlation between the furosemide dose and the change in Prop_Urea+/eOSM_ during the same period (*r* = 0.61, *P* < .001). Prop_Urea+/eOSM_ ICU days 3–7 increased in patients treated with furosemide and decreased in those who were not (Figure 4B). The correlation between Prop_Urea+/eOSM_ and eOSM on ICU day 7 adjusted for cumulative furosemide dose ICU days 3–7 and eOSM was *r* = 0.55. In patients with and without RRT the correlation between Prop_Urea+/eOSM_ ICU day 7 and eOSM was *r* = 0.54 and *r* = 0.55, respectively. In the subgroup of patients not administered furosemide and not treated with RRT during ICU stay (*n* = 17) there was a strong correlation between eOSM and Prop_Urea+/eOSM_ ICU day 7 (*r* = 0.92). For patients treated with furosemide, but not treated with RRT the correlation between Prop_Urea+/eOSM_ and eOSM on ICU day 7 was *r* = 0.61. Only four patients were treated with RRT, but not treated with furosemide the correlation between Prop_Urea+/eOSM_ and eOSM on ICU day 7 was *r* = 0.94.

There was no difference in eOSM in patients who survived to 90 d post ICU admission compared to those who did not survive (Figure S1). However, Prop_Na+/eOSM_ was higher and Prop_Urea+/eOSM_ was lower during ICU days 3–7 in survivors compared to nonsurvivors (Figure 1). Prop_Urea+/eOSM_ was not a predictor of 90 date mortality on ICU day 7 (Odds ratio 1.3×10^6^ (0.71–2.6 × 10^12^,  *P* = .06).

In the sensitivity analysis the correlation between eOSM and Prop_Na+/eOSM_, Prop_K+/eOSM_, Prop_Urea/eOSM_, Prop_Glucose/eOSM_ with imputed data (Table S2) was similar to that in Table 2.

## Discussion

### Key Findings

In a cohort of stabilized ICU patients, we found that Prop_Urea/eOSM_ increases during body volume water reduction and with increasing eOSM there is a shift of osmolytes from Na^+^ and K^+^ to urea, ie from nonorganic to organic osmolytes. This is also present in patients with normal eGFR and when the model was adjusted for eGFR. Additionally, we found that changes in urea and Prop_Urea/eOSM_ were associated with eGFR, but very weakly with nitrogen, fat and energy fluid administration. Changes in Prop_Urea/eOSM_ during fluid volume reduction in ICU patients were associated with fluid balance and furosemide treatment. Finally, although the level and the evolution of eOSM during ICU days 3–7 was similar in survivors and non-survivors, Prop_Urea/eOSM_ was higher in patients who did not survive to 90 d after ICU admission.

The increasing plasma osmolality in ICU patients in the de-resuscitation phase is generally considered a passive process due to free water loss.^22^ In this study, we show that the Prop_Urea/eOSM_ increases during ICU stay and with increasing estimated osmolality, suggesting that urea production increases and/or urea elimination decreases in these patients. However, this phenomenon is not related to urine output and with water and sodium loss. Furthermore, as it is present even when adjusted for renal function and in patients with normal renal function, urea production seems to be the main contributor for this change from ionic to nonionic osmolytes. Accordingly, our data suggest that patients that have negative fluid balance and increase in osmolality, ie get dehydrated, also become catabolic. The source of urea is protein degradation primarily from muscle wasting, occurring already during the first ICU days.^23^

Many patients in the ICU in the postresuscitative phase achieve negative fluid balance either spontaneously or with active fluid volume reduction and their caloric and protein needs are not always met by the administered nutrition. The processes of adaption to water and energy shortage is not an isolated process in ICU patients and have similarities with *aestivation*, an evolutionary conserved survival strategy among many invertebrates and vertebrates. Arid conditions that restrict food and water availability are the common triggers for aestivation, often but not always triggered by elevated ambient temperatures. Several complex physiologic and metabolic adjustments are activated to retain water in the body to prevent severe dehydration. Increase in organic osmolyte production is coupled with increased transporter-driven urea osmolyte accumulation in the skin and renal barriers to limit transepithelial water loss.^18^ Other key features include a strong suppression of metabolic rate,^24^ altered nitrogen metabolism and mechanisms to preserve and stabilize organs, cells, and macromolecules over many weeks or months of dormancy.^25^

Plasma urea levels and Prop_Urea/eOSM_ in our cohort were not linked to nitrogen administration. These findings suggest that the increase in plasma urea was not due to protein overload, and that the protein breakdown was not affected by protein administration. On other the hand, Prop_Urea/eOSM_ was higher in patients with a negative fluid balance compared to in those with a positive fluid balance and there was a weak but consistent association between plasma urea vs. fat and energy administration. While the former finding points to that loss of water increases the role urea as osmolyte, the latter findings, given the weak association, be a result of several factors increasing protein turnover.

Recently, the phenomenon of aestivation has gained attention in other areas such as in psoriatic mice with severe cutaneous water loss. In response to this dehydration stress, the mice activate aestivation-like water conserving motifs to maintain their body hydration status. Besides efficient transporter-mediated osmolyte accumulation in the epithelial kidney barrier, successful water conservation also requires increased urea osmolyte synthesis in the liver. Although our data do not allow assessment of urea cycle activity, the increase in Prop_Urea/eOSM_ independent of renal function or nitrogen administration could suggest an aestivation induced water conservation mechanism. The increased production of urea and other organic osmolytes is energy intense and requires utilization of endogenous amino acids stored in muscles. Urea levels increased with increased energy and fat administration to patients in our study which could suggest that noncarbonhydrate fuel allows for increased gluconeogenesis.

We also report that Prop_Urea/eOSM_ increased with increased furosemide use. Since furosemide increases both renal water, sodium and potassium losses, the increase in urea as an osmolyte is expected. However, extended use of loop diuretics have been associated with muscle wasting in patients with renal and liver failure.^26,27^ The association between eOSM and Prop_Urea/eOSM_ was present also after adjustment for furosemide dose, and in patients with and without RRT. In the 17 patients treated neither with diuretics nor RRT during the study there was a very strong correlation between eOSM and Prop_Urea/eOSM_. These findings suggest that the change to nonionic osmolytes in our cohort is not an effect of diuretic use only. If aestivation mechanisms are activated in ICU patients, and furosemide use enhances these mechanisms, furosemide use could lead to increased muscle breakdown.

Accelerated muscle wasting could be one possible explanation why Prop_Urea/eOSM_ is higher in patients that do not survive to 90 d compared, to those who do in our cohort. Physiological stress from critical illness increases serum cortisol concentrations^28^ and in some ICU cohorts patients with higher endogenous plasma cortisol levels have a higher survival rate than those with lower levels.^29^ Based on our findings future studies could investigate if patients with higher serum cortisol levels loose less sodium in their urine, leading to a lower level of protein degradation from muscle.

### Strengths and Limitations

As far as we know, this is the first study to assess the evolution of ionic and nonionic osmolytes in ICU patients and coupling it to a possible aestivation-like reaction. Secondly, our study included a large number of patients with high-resolution data. Finally, the multicenter design decreases the risk of selection bias and increases the external validity of the findings.

The study also has limitations due to the retrospective design, but data was systematically gathered, decreasing the effect of this. Being a posthoc analysis based on findings from our previous study, we were not able to assess urea synthesis and metabolism as well as protein synthesis and protein breakdown in the patients.

A further limitation of the study is that we used standard blood gas analysers to measure sodium and potassium. This method is reasonably accurate for clinical decision-making, but is not considered to be the gold standard method. Yet, the key findings of the study are solid and consistent with data from laboratory studies.

Additionally, we lack data on explanatory variables such as urine urea, as well as the extent of muscle wasting since these are not routinely measured in ICU patients. Moreover, glomerular filtration rate was estimated, according to the routines of the Swedish hospitals, from an endogenous marker.

Finally, we used eOSM instead of measured osmolality. The main reason for this is the retrospective design in this posthoc study. Studies in this area suggest that the formula used for calculation of estimated osmolality in our study is the one considered to be most accurate compared to measured serum osmolality in ICU patients and that deviations between these is minor.^7^

### Clinical Implications

Our data suggest that body water volume reduction could potentially accelerate muscle wasting in ICU patients, a finding that might affect the way we treat patients during the fluid volume reduction phase. If confirmed in future studies free water administration to target normal osmolality, or less aggressive fluid volume reduction, might limit the process of muscle wasting in ICU patients.

Our study also demonstrates that urea does not increase with increased nitrogen load, ie substrate mobilization from the muscles is triggered by critical illness irrespective of nitrogen administration in the quantities currently used in Swedish ICUs. This finding might affect the way we think regarding nutritional aspects in critically ill patients.

Finally, our study suggests that increase in urea is not entirely a result of decreased eGFR. This means that initiating RRT triggered by isolated plasma urea levels could in theory lead to RRT despite acceptable GFR.

### Future Studies

Given the findings in our study, further clinical studies on how protein metabolism as well as the urea cycle is affected by dehydration are warranted. Prospective randomized studies on the effect of free water administration to target normal osmolality or less aggressive fluid volume reduction in ICU patients on the level of muscle wasting are also of interest.

### Conclusions

In ICU patients in the postresuscitation phase, we found that Prop_Urea/eOSM_ increases during fluid volume reduction and with increasing eOSM, statistically independently of nitrogen administration and renal function. This a shift of from ionic osmolytes to urea during fluid volume reduction has similarities to that seen in aestivating animals.

## Authors' Contributions

SN, JT, MH, RF, and ML contributed to conception and design of the study. SN and AB collected patient data. SN, MH, and ML performed data analysis. The manuscript was drafted by SN, JT, MH, RF, and ML. All authors commented on previous versions of the manuscript. All authors read and approved the final manuscript for publication.

## Supplementary Material

zqab055_Supplemental_File_urea_rev_1

## Data Availability

The data underlying this article cannot be shared publicly due to the Swedish regulations on sensitive data. The data will be shared on reasonable request to the corresponding author.
