# Supplementary material for: The Contribution of Plasma Urea to Total Osmolality During Iatrogenic Fluid Reduction in Critically Ill Patients
Source: Function (Oxf). 2021 Oct 29;3(1):zqab055. doi: 10.1093/function/zqab055 (PMC8788870; doi:10.1093/function/zqab055)
Supplement: zqab055_Supplemental_File_urea_rev_1 [file zqab055_supplemental_file_urea_rev_1.docx]

Supplemental data


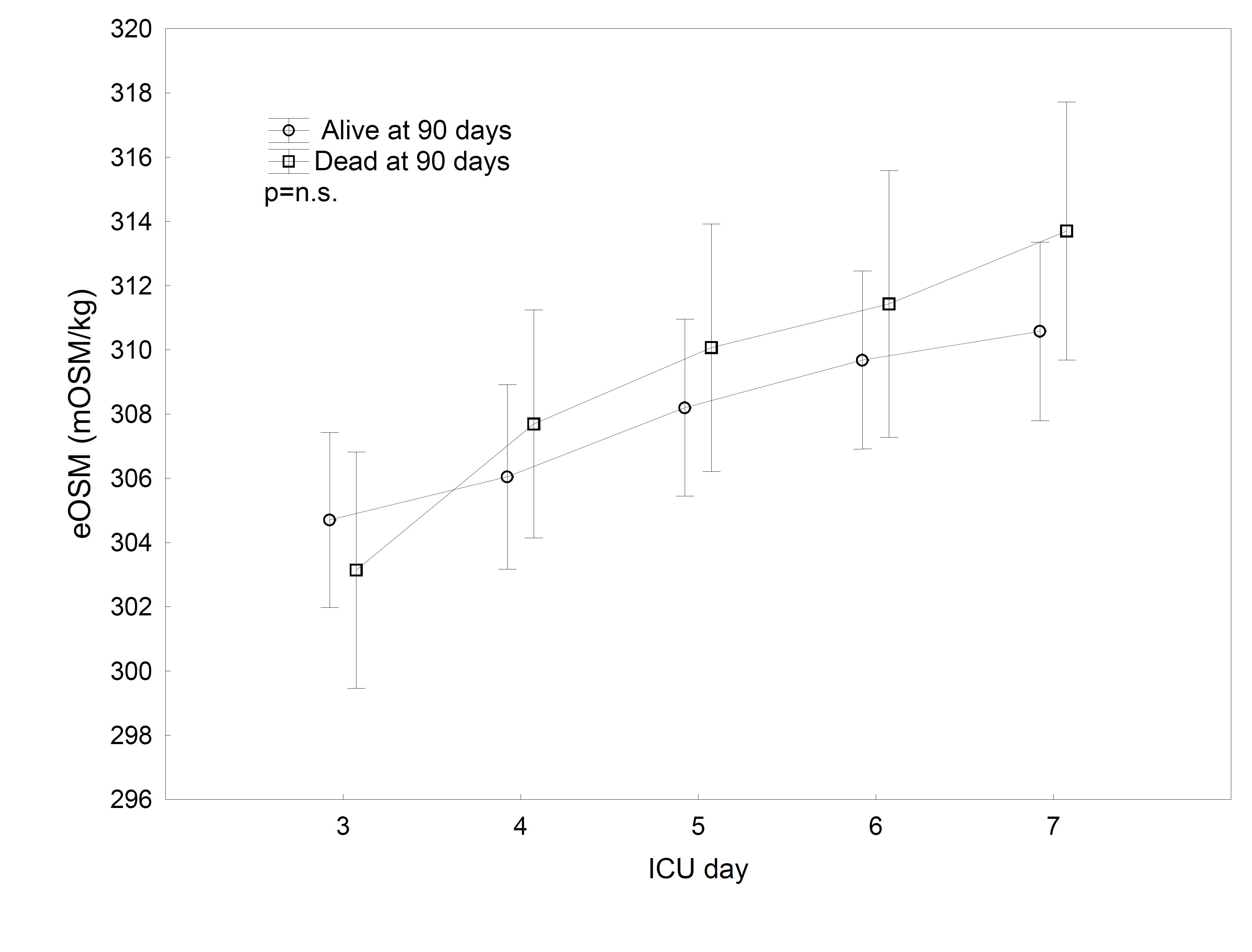


Figure S1. The evolution of estimated serum osmolality (eOSM) on ICU days 3 to 7 in survivors and non-survivors at day 90 post ICU admission. Mean ± standard error of the mean.

Table S1. Laboratory data at admission and during the 5-day observation period in the ICU. Data are presented as mean ± SD.

|  | ICU Admission | ICU Day 3 | ICU Day 4 | ICU Day 5 | ICU Day 6 | ICU Day 7 |
| --- | --- | --- | --- | --- | --- | --- |
| Blood haemoglobin, g dl^-1^ | 11.0 (9.4-12.9) | 9.8 (8.7-11.0) | 9.5 (8.6-10.8) | 9.5 (8.6-10.6) | 9.6 (8.7-10.7) | 9.6 (8.5-10.7) |
| Plasma sodium, mmol l^-1^ | 137 (134-140) | 138 (135-141) | 139 (137-143) | 140 (138-143) | 140 (137-144) | 141 (137-144) |
| Plasma potassium, mmol l^-1^ | 4.1 (3.7-4.5) | 4.1 (3.9-4.2) | 4.1 (3.8-4.4) | 4.1 (3.8-4.4) | 4.1 (3.8-4.3) | 4.2 (3.9-4.4) |
| Plasma creatinine, µmol l^-1^ | 111 (72-188) | 110 (74-166) | 99 (75-148) | 95 (68-146) | 92 (68-142) | 91 (62-145) |
| Plasma urea, mmol l^-1^ | 10.1 (5.7-17) | 8.7 (5.3-13) | 10 (6.2-33) | 8.8 (6-14) | 9.7 (7.1-15) | 11.1 (7.6-16) |
| GFR, ml min^-1^ | 49 (27-78) | 51 (30-79) | 60 (35-90) | 61 (35-85) | 62 (39-85) | 63 (38-88) |
| Blood glucose, mmol l^-1^ | 8.5 (6.6-10.8) | 7.8 (6.9-9.6) | 7.7 (6.7-9.3) | 7.7 (6.7-9) | 7.8 (6.7-10.7) | 7.8 (7.0-9.3) |

eGFR, estimated glomerular filtration rate.

Table S2. Correlations between the proportions of plasma sodium (Na+), potassium (K+), urea and glucose (Prop) to the estimated serum osmolality (eOsm) and eOSM on day 3 and 7 in the intensive care unit (ICU) with imputed values.

|  | ICU Day 3 | ICU Day 4 | ICU Day 5 | ICU Day 6 | ICU Day 7 |
| --- | --- | --- | --- | --- | --- |
| Prop_Na+/eOSM_ vs eOSM | -0.56*** | -0.69*** | -0.71 | -0.68*** | -0.60*** |
| Prop_K+/eOSM_ vs eOSM | -0.21*** | -0.24*** | -0.24*** | -0.21*** | -0.31*** |
| Prop_Urea/eOSM_ vs eOSM | 0.60*** | 0.72*** | 0.74*** | 0.71*** | 0.67*** |
| Prop_Glucose/eOSM_ vs eOSM | 0.02 | 0.08 | -0.08 | -0.10 | -0.18* |
